# Missing phonemes are perceptually restored but differently by native and non-native listeners

**DOI:** 10.1186/s40064-016-2479-8

**Published:** 2016-06-14

**Authors:** Mako Ishida, Takayuki Arai

**Affiliations:** Faculty of Science and Technology, Sophia University, 7-1, Kioi-cho, Chiyoda-ku, Tokyo, 102-8554 Japan

**Keywords:** Phonemic restoration, Perceptual restoration, Speech perception, Robustness, Bottom-up processing, Top-down processing, L1 listening, L2 listening

## Abstract

This study investigates how similarly present and absent English phonemes behind noise are perceived by native and non-native speakers. Participants were English native speakers and Japanese native speakers who spoke English as a second language. They listened to English words and non-words in which a phoneme was covered by noise (added; phoneme + noise) or replaced by noise (replaced; noise only). The target phoneme was either a nasal (/m/ and /n/) or a liquid (/l/ and /r/). In experiment, participants listened to a pair of a word (or non-word) with noise (added or replaced) and a word (or non-word) without noise (original) in a row, and evaluated the similarity of the two on an eight-point scale (8: very similar, 1: not similar). The results suggested that both native and non-native speakers perceived the ‘added’ phoneme more similar to the original sound than the ‘replaced’ phoneme to the original sound. In addition, both native and non-native speakers restored missing nasals more than missing liquids. In general, a replaced phoneme was better restored in words than non-words by native speakers, but equally restored by non-native speakers. It seems that bottom-up acoustic cues and top-down lexical cues are adopted differently in the phonemic restoration of native and non-native speakers.

## Background

Phonemic restoration is a phenomenon in which a person hears an illusory sound of a missing phoneme as if it is present. Warren (1970) firstly demonstrated phonemic restoration by using a sentence, “The state governors met with their respective legislatures convening in the capital city”, in which the first phoneme “s” in “legislatures” was completely deleted (along with the adjacent acoustic segments of “s”) and replaced by a cough. In his study, participants were not only unable to detect the exact location of the cough, but even responded that all the speech sounds were present. It seems that the deleted “s” was perceptually restored and perceived as if it was there. In fact, phonemic restoration takes place very often in our life. When two people communicate outside, for example, in the presence of extraneous sounds such as trains, cars, or public announcements, they need to restore the interrupted, distorted, or masked speech to understand each other (Broadbent 1958; Cherry 1953; Cherry and Wiley 1967; Warren 1970; Warren and Warren 1970; Warren and Obusek 1971). For communication, listeners not only allocate their attention to specific speech signals and extract messages out of noise (cocktail party effects), but they also perceptually restore the distorted or missing portion of speech (perceptual restoration). Warren (1970) reported that perceptual restoration takes place even when the replacing sound is changed to tones, buzzes, or noise. The missing segment of speech can be restored, under certain conditions (Kashino 2006), when the deleted segment is covered by extraneous sounds.

In fact, the restorability of a missing phoneme follows the ‘masking potential rule’ (Kashino 2006). That is, the deleted phoneme is perceptually restored when the replacing sound has the sufficient spectral, temporal, and spatial characteristics to mask the deleted phoneme. Warren and Warren (1970) reported that the deleted phoneme was perceptually restored when the replacing sound was as loud as or louder than the original phoneme, and when the replacing and replaced sound had the same center frequency. Samuel (1981a, b) also reported that the restorability of a missing phoneme depends on the acoustic similarity between the replacing and replaced sound; stop and fricative consonants were restored mostly when replaced by white noise (ceiling effect), while nasal and liquid consonants were restored about by half, and vowels were least restored (floor effect). This result was reversed when the replacing sound was changed from white noise to pure tone; vowels were mostly restored when replaced by pure tone, while nasal and liquid consonants were restored about by half, and stop and fricative consonants were least restored. Samuel (1981b) also reported that a missing phoneme, in general, was restored better when replaced by white noise than pure tone. The acoustic similarity between the target phoneme and the replacing sound accelerates perceptual restoration, and speech sounds are better restored when replaced by white noise.

The perceptual restoration of a missing phoneme also depends on contextual information. Warren and Obusek (1971) reported that listeners restored a missing phoneme based on the subsequent context. When listeners listened to a sentence, “There was time to (?)ave….”, for example, and when the rest of the speech was talking about a friend who was departing, listeners tended to restore the target segment as “wave”, while the segment could be any words as “save”, “shave”, or “rave”. Samuel (1981a, b, 1996) also reported that a missing phoneme in words was better restored than that in non-words; a missing phoneme /r/ in “recovery”, for example, was better restored than that in “stroppuvvery”. Additionally, the illusory sound of a missing phoneme was perceived only when the replaced segment was presented in lexical items, and the illusory sound was not perceived when the replaced segment was presented in isolation. Phonemic restoration seems to take place when lexical context is available (Ganong 1980; Warren and Sherman 1974; Warren and Warren 1970). In addition, Warren and Obusek (1971) suggested that phonemic restoration also depends on listeners’ language proficiency. If listeners are familiar with the target language (i.e., phonemes, phonotactics, vocabulary, semantics, and pragmatics), a missing phoneme can be easily and inattentively restored. Therefore, native speakers have presumably more advantages in phonemic restoration as compared to non-native speakers, as native speakers have a vast amount of intuitive and acquired linguistic knowledge.

As was suggested above, phonemic restoration takes place by integrating bottom-up acoustic cues and top-down contextual cues. However, how these cues are processed, combined, and utilized (which is often described in speech perception models such as TRACE, Motor Theory, or COHORT) is still not clear, while it is likely that language proficiency is somewhat involved. Kashino and Craig (1994) suggested that non-native speakers with advanced proficiency are more successful in perceptual restoration than those with limited proficiency. Moreover, advanced learners seem to anticipate upcoming speech signals even prior to the onset of speech. Advanced learners are presumably more adept at effectively combining bottom-up acoustic cues and top-down contextual cues to make sense of distorted speech. It is likely that the use of linguistic knowledge is essential for successful perceptual restoration. However, at the same time, linguistic knowledge can also lead to failures in perceptual restoration (slips of the ear), especially in the cross-language domain (Bond 1999; Voss 1984). For example, Kashino and Craig (1994) reported that Japanese native speakers who spoke English as a second language tended to misperceive a word “hardware” as “haraware”, and “stairway” as “stayaway”, with a vowel insertion in consonant clusters, possibly because Japanese language has a CV (consonant + vowel) as a basic linguistic unit, and this anticipatory rule was applied in perception of English. In addition, Kashino et al. (1992) reported that the phonotactic rules of listeners’ mother tongue affected the perceptual restoration: While both Dutch and Japanese native speakers restored the intervocalic voiceless stop consonant in a VCV segment (vowel + consonant + vowel) by utilizing co-articulatory cues of VC transition (syllable-final) as well as CV transition (syllable-initial), Dutch listeners were more adept at utilizing the VC transition to restore the voiceless stop consonant. This is possibly because Dutch listeners have both VC and CV as a basic linguistic unit in their mother tongue, while Japanese listeners have only CV as a basic linguistic unit. It seems that the phonotactic constraints of listeners’ mother tongue affects the perceptual restoration, and top-down contextual cues and bottom-up acoustic cues are combined differently among native and non-native speakers.

While phonemic restoration is a strong auditory illusion and a missing phoneme can be perceptually restored, listeners also differentiate the presence and absence of a phoneme behind noise. When listeners hear words and non-words in which a phoneme was covered by noise (added; phoneme + noise) or replaced by noise (replaced; noise only), they seem to perceive the difference (Ishida and Arai 2015; Samuel 1981a, b, 1996; Mattys et al. 2014). When listeners listen to a pair of a word with noise (added or replaced) and a word without noise (original) in a row, and evaluate the similarity of the two on an eight-point scale (8: very similar, 1: not similar), they respond that the added phoneme was more similar to the original sound than the replaced phoneme to the original sound (Ishida and Arai 2015; Mattys et al. 2014; Samuel 1996). Listeners seem to instantly and inattentively perceive the subtle difference between the presence and absence of acoustic signals behind noise, despite the fact that the missing phoneme is often perceptually restored. It is possible that this bottom-up acoustic processing is, in part, an independent process which does not interact with linguistic factors.

The current study explores how bottom-up acoustic cues and top-down lexical cues are combined for phonemic restoration, with a special focus on the following three factors: (1) Noise (added vs. replaced), (2) Phonemes (nasal vs. liquid), and (3) Lexical knowledge (word vs. non-word). This study especially attempts to explore how phonemic restoration is realized in relation to listeners’ language proficiency, i.e., first (L1) and second language (L2). There are three research questions: (1) How similarly present and absent phonemes behind noise (i.e., original waveforms present and absent behind noise) are perceived in L1 and L2? (2) Is the restorability of missing phonemes shared among native and non-native speakers, regardless of L1 phonemic inventory? (3) How much lexical information contributes to the restoration of missing phonemes in L1 and L2?—Is the missing phoneme in words better restored than that in non-words in both L1 and L2? The first research question examines the perceptual sensitivity to the presence and absence of phonemes behind noise in L1 and L2, to see if the bottom-up acoustic processing at the very beginning of speech perception is independent of linguistic factors, and, therefore, shared among native and non-native speakers. This question examines if there is an acoustic processing which does not interact with or affected by linguistic factors. The second research question examines the restorability of phonemes which exist and do not exist in listeners’ mother tongue, to see if the L1 linguistic factor is involved in phonemic restoration. This question examines how phonemes are processed when deleted, and if the deleted part of speech is processed just as an acoustic entity (therefore, similar perceptual restoration among native and non-native speakers) or as a linguistic entity (therefore, native speakers have more advantages over non-native speakers). The third research question examines if lexical context supports phonemic restoration in L1 and L2, and if lexical advantages over nonsense words are shared among native and non-native speakers. This question examines how much lexical knowledge contributes to phonemic restoration. The current study attempts to explore how bottom-up acoustic cues and top-down lexical cues interact and support phonemic restoration in L1 and L2, in search of speech perception mechanisms, and educational implications for L2 listening enhancement.

## Results

The perceptual similarity of the ‘added’ phoneme (phoneme + noise) to the original sound, and the perceptual similarity of the ‘replaced’ phoneme (noise only) to the original sound were evaluated on an eight-point scale (8: very similar, 1: not similar). A subject-wise analysis was performed to see any difference between native and non-native speakers. Table 1 shows the mean similarity scores of English native speakers (NS) (*N* = 30) and Japanese native speakers who spoke English as a second language (NNS) (*N* = 30). The mean similarity score was computed for eight different conditions: 2 noise conditions (added vs. replaced) × 2 phonemic conditions (nasal vs. liquid) × 2 lexical conditions (word vs. non-word). The mean was firstly computed for each subject, by computing their average scores over thirty trials per condition, and the grand mean was computed for each condition. The marginal mean was also computed and displayed in Table 1.

**Table 1:** The mean similarity scores on an eight-point scale

	Words	Non-words	Marginal mean
*NS*			
Added			
Nasal	7.01	6.81	6.91
Liquid	6.89	6.67	6.78
Marginal mean	6.95	6.74	
Replaced			
Nasal	6.32	6.09	6.21
Liquid	6.14	5.51	5.83
Marginal mean	6.23	5.80	
*NNS*			
Added			
Nasal	6.36	6.44	6.40
Liquid	6.18	6.19	6.18
Marginal mean	6.27	6.32	
Replaced			
Nasal	5.85	5.84	5.85
Liquid	5.41	5.29	5.35
Marginal mean	5.63	5.57	

*NS* native speakers of English, *NNS* non-native speakers of English (Japanese native speakers of English who spoke English as a second language

**Fig. 1:**
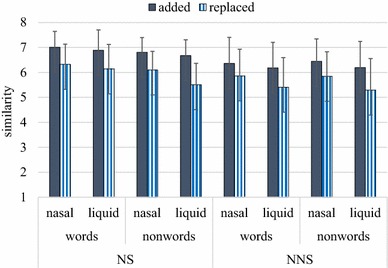
The mean similarity scores on an eight-point scale, with *error bars* representing standard deviations. The scores were evaluated by English native speakers (NS) and non-native speakers (NNS)

The restorability of a missing phoneme was observed in the difference between the ‘added’ and ‘replaced’ scores. When the difference between the added (original phoneme behind noise) and replaced scores (*NO* original phoneme behind noise) is small, it means, that the present and absent phonemes were equivalently perceived. When the difference between the added and replaced scores is big, it means, that the present and absent phonemes were differently perceived. Figure 1 shows the average scores in bar graphs, with error bars representing the standard deviation. In general, the ‘added’ phoneme (phoneme + noise) yielded higher similarity scores than the ‘replaced’ phoneme (noise only). It seems that the present phoneme in the added condition was differentiated from the absent phoneme in the replaced condition, while the absent phoneme was perceptually restored and perceived as if it was present.

**Fig. 2:**
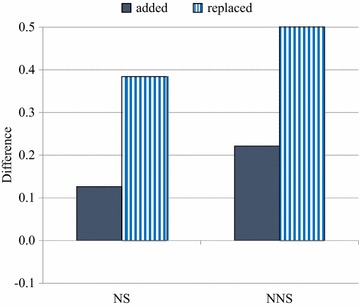
The difference between the similarity scores of nasals and liquids. Computed as “nasals (added) minus liquids (added)” on the *left* in the *solid bar* and “nasals (replaced) minus liquids (replaced)” on the *right* in the *stripe bar* for native (NS) and non-native speakers (NNS)

Figure 2 shows the difference between the similarity scores of nasals and liquids, computed as “nasals (added) minus liquids (added)” in the solid bar, and “nasals (replaced) minus liquids (replaced)” in the stripe bar for both native and non-native speakers. The results suggest that the difference between nasals (added) and liquids (added) was .13 for native speakers and .22 for non-native speakers, while the difference between nasals (replaced) and liquids (replaced) was .38 for native speakers and .50 for non-native speakers. The positive value in Fig. 2 indicates the greater perceptibility (added) and restorability (replaced) of nasals than liquids. It seems that both native and non-native speakers perceived the added and replaced nasals more than liquids, but the perception of nasals, in general, was greater among non-native speakers than native speakers. Additionally, a paired *t* test was carried out with the similarity scores of nasals (added) of native and non-native speakers, and with the similarity scores of liquids (added) of native and non-native speakers, to see whether or not the perceptibility of phonemes behind noise (added) is meaningfully different among native and non-native speakers. The results suggested that native speakers (*M* = 6.91, *SD* = .56) perceived nasals behind noise (added) significantly more than non-native speakers (*M* = 6.40, *SD* = .90), *t* (29) = 3.14, *p* = .004, and native speakers (*M* = 6.78, *SD* = .68) perceived liquids behind noise (added) significantly more than non-native speakers (*M* = 6.18, *SD* = .97), *t* (29) = 3.30, *p* = .003. In general, native speakers perceived phonemes behind noise (added) significantly more than non-native speakers. In addition, another paired *t*-test was carried out with the similarity scores of nasals (replaced) of native and non-native speakers, and with the similarity scores of liquids (replaced) of native and non-native speakers, to see whether or not the restoration size of native and non-native speakers was meaningfully different. The results suggested that native speakers (*M* = 6.21, *SD* = .67) restored missing nasals (replaced) greatly more than non-native speakers (*M* = 5.85, *SD* = .98), *t* (29) = 1.89, *p* = .069, and native speakers (*M* = 5.83, *SD* = .79) restored missing liquids (replaced) greatly more than non-native speakers (*M* = 5.35, *SD* = 1.15), *t* (29) = 1.98, *p* = .058. While nasals (replaced) are restored more than liquids (replaced) in general, the restoration size of native speakers seems to be bigger than that of non-native speakers. Taken together, native speakers were significantly better than non-native speakers at perceiving speech signals behind noise (added) and greatly better at restoring missing phonemes behind noise (replaced), while both native and non-native speakers perceived nasals more than liquids.

**Fig. 3:**
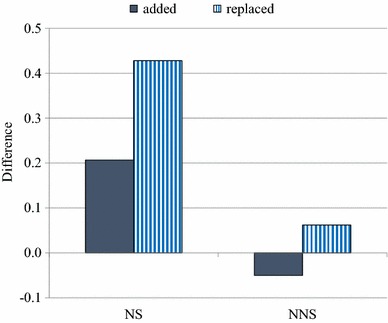
The difference between the similarity score of words and non-words. Computed as “words (added) minus non-words (added)” on the *left* in the *solid bar* and “words (replaced) minus non-words (replaced)” on the *right* in the *stripe bar* for native (NS) and non-native speakers (NNS)

Figure 3 shows the difference between the similarity scores of words and non-words, computed as “words (added) minus non-words (added)” in the solid bar, and “words (replaced) minus non-words (replaced)” in the stripe bar for both native and non-native speakers. The results suggest that the difference between words (added) and non-words (added) was .21 for native speakers and −.05 for non-native speakers. On the other hand, the difference between words (replaced) and non-words (replaced) was .43 for native speakers and .06 for non-native speakers. The positive value in Fig. 3 indicates the lexical influence in perception of the existing (added) and non-existing (replaced) phoneme behind noise. It seems that native speakers perceived the existing phoneme behind noise (added) slightly better in *words* than non-words, while non-native speakers perceived the existing phoneme behind noise (added) slightly better in *non*-*words* than words. In addition, native speakers perceptually restored the non-existing phoneme (replaced) better in words than non-words, while non-native speakers restored the non-existing phoneme (replaced) in words and non-words equivalently. There seems to be lexical influence in perception of the existing and non-existing phoneme among native speakers, but little influence among non-native speakers. It seems that lexical context helped the perceptual sensitivity (added) and phonemic restoration (replaced) of native speakers, while it did not help those of non-native speakers.

An ANOVA was firstly carried out respectively for English native speakers and non-native speakers, with two noise factors (added vs. replaced), two phonemic factors (nasal vs. liquid), and two lexical factors (word vs. non-word) as within-subject factors. As for native speakers, the added sound was perceived significantly more similar to the original sound than the replaced sound to the original sound, *F* (1, 29) = 66.75, *p* < .001. A nasal consonant in noise was perceived significantly more similar to the original sound than a liquid consonant in noise to the original sound, *F* (1, 29) = 33.00, *p* < .001. The target sound in words was perceived significantly more than the target sound in non-words, *F* (1, 29) = 8.39, *p* = .007. There was a significant two-way interaction between noise and phonemic factors, *F* (1, 29) = 15.21, *p* = .001, a significant two-way interaction between noise and lexical factors, *F* (1, 29) = 3.96, *p* = .056, and a significant two-way interaction between phonemic and lexical factors, *F* (1, 29) = 11.48, *p* = .002, and a significant three-way interaction among noise, phonemic, and lexical factors, *F* (1, 29) = 11.03, *p* = .002. As for non-native speakers, the added sound was perceived significantly more similar to the original sound than the replaced sound to the original sound, *F* (1, 29) = 38.15, *p* < .001. A nasal consonant in noise was perceived significantly more similar to the original sound than a liquid consonant in noise to the original sound, *F* (1, 29) = 38.66, *p* < .001. On the other hand, the target sound in words and non-words were perceived equivalently, *F* (1, 29) = .01, *p* = .95. There was a significant two-way interaction between noise and phonemic factors, *F* (1, 29) = 9.07, *p* = .005, but no significant two-way interaction between noise and lexical factors, *F* (1, 29) = 2.23, *p* = .15, and no significant two-way interaction between phonemic and lexical factors, *F* (1, 29) = 2.15, *p* = .15, and no significant three-way interaction among noise, phonemic, and lexical factors, *F* (1, 29) = .03, *p* = .86. Taken together, both native and non-native speakers perceptually differentiated the added and replaced sound. In addition, both native and non-native speakers perceived the nasal in noise significantly more similar to the original sound than the liquid in noise to the original sound (a significant two-way interaction between noise and phonemes among both native and non-native speakers). However, while native speakers perceived the target sound in words better than the target sound in non-words, non-native speakers perceived the target sound in words and non-words equivalently (a significant two-way interaction between noise and lexicality for native speakers, but no significant two-way interaction for non-native speakers). It seems that the lexicality supported the perception of native speakers, while lexicality did not support the perception of non-native speakers.

Additionally, an ANOVA was carried out with two language groups as between-subject factors (native vs. non-native), and two noise factors (added vs. replaced), two phonemic factors (nasal vs. liquid), and two lexical factors (word vs. non-word) as within-subject factors. The results suggested that the speech perception of native speakers was significantly different from that of non-native speakers, *F* (1, 58) = 5.70, *p* = .02. However, as was suggested in the previous paragraphs, native and non-native speakers share some commonalities. The added phoneme was perceived significantly more similar to the original sound than the replaced phoneme to the original sound by both native and non-native speakers, *F* (1, 58) = 101.11, *p* < .001; there was no two-way interaction between noise and language factors, *F* (1, 58) = .75, *p* = .39. In addition, a nasal consonant in noise was perceived significantly more similar to the original sound than a liquid consonant in noise to the original sound by both native and non-native speakers, *F* (1, 58) = 71.11, *p* < .001; there was no two-way interaction between phonemic and language factors, *F* (1, 58) = 1.96, *p* = .17. On the other hand, while the target sound in words was perceived significantly more similar to the original sound than the target sound in non-words to the original sound, *F* (1, 58) = 3.80, *p* = .056, there was a slight two-way interaction between lexical and language factors, *F* (1, 58) = 3.46, *p* = .068. As was suggested in the respective ANOVA in the previous paragraph, this would suggest that lexical context supported the perceptual sensitivity (added) and phonemic restoration (replaced) of native speakers, while it did not support those of non-native speakers. As for interactions, there was a significant two-way interaction between noise and phonemic factors, *F* (1, 58) = 22.11, *p* < .001, and no significant three-way interaction among noise, phonemic, and language factors, *F* (1, 58) = .06, *p* = .82. There was also a significant two-way interaction between noise and lexical factors, *F* (1, 58) = 6.17, *p* = .016, and no significant three-way interaction among noise, lexical, and language factors, *F* (1, 58) = .68, *p* = .41, while the respective ANOVA in the previous paragraph showed a significant interaction between noise and lexical factors for native speakers, and no significant interaction for non-native speakers. There was also a significant two-way interaction between phonemic and lexical factors, *F* (1, 58) = 11.79, *p* = .001, and no significant three-way interactions among phonemic, lexical, and language factors, *F* (1, 58) = 1.86, *p* = .18, while the respective ANOVA in the previous paragraph showed a significant two-way interaction between phonemic and lexical factors for native speakers and no-significant interaction for non-native speakers. On the other hand, there was a significant three-way interaction among noise, phonemic, and lexical factors, *F* (1, 58) = 5.13, *p* = .027, and a significant four-way interaction among noise, phonemic, lexical, and language factors, *F* (1, 58) = 3.94, *p* = .052.

As a whole, both native and non-native speakers perceived the difference between the ‘added’ (phoneme + noise) and ‘replaced’ sound (noise only). In addition, both native and non-native speakers perceived the present and absent nasals significantly more than liquids. The only difference between native and non-native speakers was the lexical support for perception; while native speakers perceived the present and absent phoneme in words better than non-words, non-native speakers perceived the present and absent phoneme in words and non-words equivalently (a significant three-way interaction among noise, phonemes, and lexicality, and a significant four-way interaction among noise, phonemes, lexicality, and language factors). Phonemic restoration seems to take place differently with the different ratio of bottom-up acoustic processing and top-down lexical processing among native and non-native speakers.

## Discussion

The current study explored how bottom-up acoustic cues and top-down lexical cues are processed, combined, and utilized in speech perception, and the results suggested that native and non-native speakers integrate the (1) noise (added vs. replaced), (2) phonemic (nasal vs. liquid), and (3) lexical factors (word vs. non-word), in part, similarly, but differently for phonemic restoration. In this study, the noise factor was examined to see the perceptual sensitivity to the presence and absence of phonemes behind noise, and how acoustic details are processed at the very beginning of bottom-up processing. The phonemic factor was examined to see how acoustic details (bottom-up processing) as well as linguistic factors (top-down processing) are integrated, and if L1 phonemic inventory was involved in this process. The lexical factor was examined to see how top-down lexical context influences phonemic restoration. The results suggested that basic bottom-up acoustic processing was relatively shared among native and non-native speakers, while there was a slight L1 influence at this stage. At the same time, native and non-native speakers were different in terms of the availability of top-down processing. The effective combination of bottom-up acoustic cues and top-down lexical cues were essential for perceptual sensitivity to speech sounds in noise (similar to cocktail party effects), and perceptual restoration of missing phonemes (phonemic restoration).

Our study suggested that one of the similarities among native and non-native speakers lay in the perceptual sensitivity to the presence and absence of phonemes behind noise (noise factor). In experiments, both native and non-native speakers perceived the ‘added’ phonemes (acoustic signals behind noise) significantly more similar to the original sound than the ‘replaced’ phonemes (*NO* acoustic signals behind noise) to the original sound. This result was also supported by previous studies with native speakers in which the presence and absence of phonemes behind noise were inattentively detected, even when listeners were engaging in cognitive tasks such as finding a particular visual pattern in the picture (Mattys et al. 2014). While the difference between the ‘added’ and ‘replaced’ sound is very subtle in hearing, the ‘added’ and ‘replaced’ sound was discriminated also in a forced choice test (Samuel 1981a, b), and on an eight-point similarity judgment task (8; very similar, 1; not similar) (Ishida and Arai 2015; Samuel, 1996). The bottom-up acoustic processing seems to be shared among native and non-native speakers, relatively independently of linguistic factors.

The restorability of a missing phoneme (phonemic factor) was also shared among native and non-native speakers. In our study, both native and non-native speakers restored missing nasals (replaced) significantly more than missing liquids (replaced). Samuel (1981a, b, 1996) reported, in his studies with native speakers, that nasals and liquids are in the medium range of restorability when replaced by white noise. The current study also supported these results, with the native speakers’ similarity score of 6.32 for nasals in words (replaced) and 6.14 for liquids in words (replaced), and 6.09 for nasals in non-words (replaced) and 5.51 for liquids in non-words (replaced). At the same time, this study suggested that nasals and liquids are also in the medium range of restorability among non-native speakers, with the similarity score of 5.85 for nasals in words (replaced) and 5.41 for liquids in words (replaced), and 5.84 for nasals in non-words (replaced) and 5.29 for liquids in non-words (replaced). There was no difference between native and non-native speakers in that the missing nasals were restored more than missing liquids. Our study suggested that the restorability of phonemes is relatively shared among native and non-native speakers, despite the fact that English native speakers had all /m/, /n/ ,/l/ , and /r/ in their L1 phonemic inventory, while Japanese native speakers only had /m/ and /n/ in their L1 phonemic inventory. It seems that missing phonemes were restored based on acoustic similarities between the replaced and replacing sound, regardless of L1 phonemic inventory. That is, the masking potential rule dominated the phonemic restoration of all listeners, and missing phonemes are processed as an acoustic entity (i.e., just as sound), rather than a linguistic entity (i.e., phonemes in a specific language). While both nasal and liquid consonants have a spectral structure of harmonics as well as formants, a nasal consonant resonates in both nasal and oral cavities, and, consequently, its oral formants are weakened as compared to a liquid consonant which has a more similar formant structure as vowels (Jakobson et al. 1952; Stevens 2000). Therefore, it can be deemed that a nasal was, relatively, acoustically closer to white noise than a liquid, and this led to the better restoration of missing nasals than missing liquids by both native and non-native speakers.

On the other hand, native and non-native speakers were different in a way that native speakers restored the replaced phonemes in words better than those in non-words, while non-native speakers restored the replaced phonemes in words and non-words equivalently (lexical factor). It seems that lexical context supported the phonemic restoration of native speakers, but not the restoration of non-native speakers (Samuel 1981a, b, 1996; Warren 1970; Warren and Warren 1970; Warren and Obusek 1971; Warren and Sherman 1974). The availability of lexical context seems to differ between native and non-native speakers, and it largely depends on listeners’ language proficiency. The vocabulary size of listeners is likely to play a significant role for phonemic restoration. In general, the average American high school graduates are deemed to have approx. 40,000–80,000 reading vocabulary in their mental lexicon (Aitchison, 2012), while how much of these are shared among non-native speakers would depend on each non-native speaker’s vocabulary size. While Hirsch and Nation (1992) suggested, in the field of second language reading, that 98–99 % coverage of text (approx. ‘one unknown word in every 50–100 running words’) is required for a good command of reading comprehension, the comparable amount of vocabulary, along with listening experience to various pronunciation, would be essential for a good command of listening comprehension. It is likely that native speakers have more lexical advantages in phonemic restoration, while second language listeners with a large vocabulary size might also enjoy lexical advantages in phonemic restoration. Expanding the vocabulary size in second language, along with listening experience, is indispensable for successful phonemic restoration.

Additionally, this study also suggested that, while native and non-native speakers were similar in a way that they could differentiate the presence and absence of acoustic signals behind noise, they were also different in a way that native speakers perceived the phonemes behind noise (added) significantly more than non-native speakers, and native speakers restored the missing phonemes (replaced) greatly more than non-native speakers. Our *t* test suggested that native speakers perceived nasals behind noise (added) significantly more than non-native speakers (*p* = .004), and native speakers perceived liquids behind noise (added) significantly more than non-native speakers (*p* = .003). At the same time, native speakers restored missing nasals (replaced) greatly more than non-native speakers (*p* = .069), and native speakers restored missing liquids (replaced) greatly more than non-native speakers (*p* = .058). This seems to suggest that the perceptibility of speech signals behind noise (added), which is similar to extracting specific speech signals out of noise (cocktail party effects), is related to listeners’ language proficiency. At the same time, the restorability of missing phonemes behind noise (replaced), which is phonemic restoration, is also related to listeners’ language proficiency. The more familiar to specific speech signals as well as speech gestures, the more perceptibility of speech sounds. Listeners might be able to perceive the target sounds if they are able to produce the target sound, as was proposed in the Motor Theory (Liberman and Mattingly 1985). It is also possible that listeners’ attention is directed to speech signals, only when listeners are familiar with the incoming speech signals, and when the signals are coded into linguistic entity in listeners’ mental lexicon (selective attention) (Broadbent 1954; Cherry 1953; Treisman 1969). With a good set of mental lexicon as well as attentional resources for articulatory gestures, native speakers might have advantages over non-native speakers in terms of the ‘perception of speech codes’ (Liberman et al. 1967). The perception and the restoration of speech signals under extraneous sounds, which occurs very often in our life, are likely to depend on listeners’ language proficiency, and, in this regard, the bottom-up acoustic processing seems to interact with linguistic factors.

As was suggested above, both native and non-native speakers shared the basic bottom-up acoustic processing; the perceptual sensitivity to the presence and absence of acoustic signals behind noise (noise factor), and the restorability of nasals in relation to liquids (phonemic factor). On the other hand, native and non-native speakers were also different in top-down processing; the better restoration of missing phonemes in words than non-words among native speakers, and the equivalent restoration of missing phonemes in words and non-words among non-native speakers (lexical factor). Additionally, as a whole, native and non-native speakers were different in a way that native speakers were better at perceiving present phonemes behind noise (added), and restoring absent phonemes behind noise (replaced) than non-native speakers. These results can be interpreted that both native and non-native speakers combine bottom-up acoustic cues and top-down lexical cues to perceive spoken messages, but the availability of top-down lexical cues is different between native and non-native speakers. Moreover, the availability of top-down processing is a critical factor for phonemic restoration. While the TRACE model of speech perception (McClelland and Elman 1986) presumes that auditory input activates lexical items in the listeners’ mental lexicon and this activation gives feedback to the phonemic layer to understand the ambiguous part of speech, it is likely that non-native speakers would struggle at the very beginning of the lexical activation because of their smaller mental lexicon. In addition, as the COHORT model of speech perception (Marslen-Wilson and Tyler 1980) suggests, listeners might also map the perceived sound onto words to understand speech, but, again, this process would be difficult for non-native speakers because of the smaller mental lexicon. The bottom-up acoustic processing and top-down lexical processing would take place among both native and non-native speakers, but the availability of top-down lexical context would differ depending on the listeners’ language proficiency.

In summary, both native and non-native speakers perceptually differentiated the presence and absence of phonemes (i.e., acoustic signals) behind noise. In addition, both native and non-native speakers restored missing nasals more than missing liquids. The difference between native and non-native speakers lay in the lexical support for the restoration of missing phonemes. In addition, the size of perception of present phonemes as well as the size of restoration of missing phonemes were bigger among native speakers than non-native speakers. These results can be understood that bottom-up acoustic processing takes place, relatively, similarly among native and non-native speakers while top-down lexical processing operates differently. Earlier studies suggested that Japanese native speakers tend to have difficulties in the discrimination of English /l/ and /r/ (bottom-up processing) as compared to English native speakers, because of the fact that Japanese language does not have /l/ and /r/ in the phonemic inventory (Goto 1971; MacKain et al. 1981; Miyawaki et al. 1975). This seems to suggest that the phonemes within the same phonemic category are easily differentiated by native speakers but not easily by non-native speakers. At the same time, the current study suggested that the phonemes in a particular phonemic category (e.g. liquids), as compared to phonemes in another phonemic category (e.g., nasals), was similarly processed among native and non-native speakers; nasal consonants were generally restored more than liquids by both native and non-native speakers. It can be understood that bottom-up processing *within* a particular phonemic category works differently among native and non-native speakers, but bottom-up processing *between* particular phonemic categories work similarly among native and non-native speakers, based on the acoustic characteristics of speech sounds.

## Conclusions

The current study attempted to explore the phonemic restoration in L1 and L2. Our study showed that (1) present and absent phonemes behind noise were similarly processed among both native and non-native speakers. That is, the presence and absence of phonemes behind noise were perceptually differentiated, although a missing phoneme was restored and perceived as if it was present. In addition, (2) the restorability of a missing phoneme was relatively equally shared among native and non-native speakers, regardless of L1 phonemic inventory. Missing nasals were restored significantly more than missing liquids, despite the fact that English native speakers had both nasals (/m/, /n/) and liquids (/l/, /r/) in their L1 phonemic inventory, while Japanese native speakers had only nasals (/m/, /n/) in their L1 phonemic inventory. The restorability of a missing phoneme seems to follow the ‘masking potential rule’. On the other hand, (3) lexical context supported the phonemic restoration of native speakers, while it did not support the phonemic restoration of non-native speakers. A missing phoneme in words was better restored than that in non-words among native speakers, while a missing phoneme was equally restored in words and non-words among non-native speakers. Additionally, as a whole, native speakers and non-native speakers were different in a way that native speakers perceived and restored the target phoneme more than non-native speakers. Taken together, missing phonemes were perceptually restored by both native and non-native speakers, and the restorability of phonemes were shared among native and non-native speakers. However, the availability of top-down processing is different between native and non-native speakers, because top-down processing largely depends on the listeners’ vocabulary size. The integration of bottom-up acoustic cues and top-down lexical cues was essential for the perception of acoustic signals as well as the restoration of missing phonemes, but how effectively listeners can integrate the bottom-up acoustic cues and top-down lexical cues would largely depend on the listeners’ language proficiency.

## Methods

### Materials

The stimuli were the same as those used by Mattys et al. (2014). There were ninety word/non-word pairs (60 test pairs + 30 filler pairs). The 60 test pairs included 30 nasal target pairs (13 /m/ and 17 /n/), and 30 liquid target pairs (15 /l/ and 15 /r/). The nasal and liquid consonants were chosen as target phonemes because the results of Samuel (1981a, b, 1996) suggested that these phonemes have a medium range of restorability when masked by white noise. The members of each word/non-word pair had the same stress pattern, and each item was comprised of four to five syllables. The members of a pair had the same last two syllables, and the phoneme before the penultimate syllable was also matched (e.g., discriminate vs. notrominate). The target phoneme was located in the first phoneme of the last syllable or was ambisyllabic between the last and penultimate syllables (e.g., discriminate vs. notrominate). The 30 filler pairs also had the same stress pattern for the word and the non-word, and every item was comprised of four to five syllables. The first syllable and the onset of the second syllable of the filler pairs were also matched. The target phoneme was located within the first syllable or at the onset of the second syllable (e.g., acknowledgmenet vs. acknallutstump). The filler pairs were included to make sure that listeners’ attention was allocated across all parts of the stimuli. All stimuli were spoken by a female native speaker of Standard Southern British English, and recorded in a sound-treated room over a cardioid dynamic headset microphone (Shure WH20, 44.1 kHz, 16-bit).

For the creation of the ‘added’ and ‘replaced’ stimuli, the target phoneme, along with the adjacent coarticulatory cues, was visually and auditorily identified. In order to make the word and non-word member of each test pair have the identical target phoneme, the members of a word/non-word pair were cross-spliced at the onset of the penultimate syllable, while all the filler pairs were not cross-spliced. The cross-splicing procedures for the test stimuli assured that every test pair would have acoustically identical last two syllables. For counterbalancing purposes, half of the splicing portion originated from words, and the other half originated from non-words. The target phoneme was then either covered by noise (‘added’) or replaced by noise (‘replaced’). For the ‘added’ stimuli, the signal-correlated noise, i.e., white noise that has the same amplitude envelope as the original acoustic waves (Schroeder 1968), was ‘added’ to the target phoneme at 0 dB SNR (added). For the ‘replaced’ stimuli, the target phoneme was replaced by the signal-correlated white noise (replaced). The mean duration of the noise was 111 ms (74–213 ms range), and the average duration of the test stimuli was 766 ms (502–1069 ms range). The stimulus set was then organized into a set of ninety words (60 test words + 30 fillers) and a set of ninety non-words (60 test non-words + 30 fillers) for the experiments.

### Participants

Thirty English native speakers from Stony Brook University in the United States (17 female, 13 male, approx. mean age 20 years old) and thirty Japanese native speakers in Japan who spoke English as a second language (18 female, 12 male, approx. mean age 28 years old) participated in this study. There was no reported hearing and speech impairment. Each individual submitted a consent form to a researcher upon the agreement to participate in this study. The consent form for English native speakers was approved by Stony Brook University Institutional Review Board (IRB), and the consent form for Japanese native speakers was approved by the Ethics Committee for ‘Research on Human Subjects’ at Sophia University.

### Procedures

This experiment had two sessions: (1) a word session and (2) a non-word session. Half of the participants were randomly assigned to the word session followed by the non-word session, and the other half was assigned to the non-word session followed by the word session, for counterbalancing purposes. Each session had 180 trials, with 90 stimuli with an ‘added’ phoneme and 90 stimuli with a ‘replaced’ phoneme. All the stimuli were presented in a random order. Each session lasted about 15 min, and the whole experiment lasted about 30–40 min.

Participants listened to the stimuli through headphones (SONY MDR-CD900ST) in a sound treated room, facing to a computer. They listened to a pair of a word (or a non-word) with noise (added or replaced) and a word (or a non-word) without noise (original) in a row, and evaluated the similarity of the two on an eight-point scale (8: very similar, 1: not similar). Listeners pressed 8 on the response pad if they clearly heard the original phoneme behind noise, and listeners pressed 1 if they least heard the original phoneme behind noise. The perceptibility of the original phoneme behind noise was evaluated from 1 to 8 in an ascending order. Participants received course credits (English native speakers) or snacks (Japanese native speakers) for their participation.
